# Higher Acid Recovery Efficiency of Novel Functionalized Inorganic/Organic Composite Anion Exchange Membranes from Acidic Wastewater

**DOI:** 10.3390/membranes11020133

**Published:** 2021-02-14

**Authors:** Muhammad Imran Khan, Abdallah Shanableh, Noureddine Elboughdiri, Karim Kriaa, Djamel Ghernaout, Saad Ghareba, Majeda Khraisheh, Mushtaq Hussain Lashari

**Affiliations:** 1 Research Institute of Sciences and Engineering, University of Sharjah, Sharjah 27272, United Arab Emirates; shanableh@sharjah.ac.ae; 2 Chemical Engineering Department, College of Engineering, University of Ha’il, P.O. Box 2440, Ha’il 81441, Saudi Arabia; ghilaninouri@yahoo.fr (N.E.); djamel_andalus@hotmail.com (D.G.); s.ghareba@uoh.edu.sa (S.G.); 3 Chemical Engineering Process Department, National School of Engineering Gabes, University of Gabes, Gabes 6011, Tunisia; kriaa_karim@yahoo.fr; 4 Chemical Engineering Department, College of Engineering, Al Imam Mohammad Ibn Saud Islamic University, Riyadh 11432, Saudi Arabia; 5 Chemical Engineering Department, Faculty of Engineering, University of Blida, P.O. Box 270, Blida 09000, Algeria; 6 Department of Chemical and Petroleum Engineering, ElMergib University, Alkhums 40414, Libya; 7 Department of Chemical Engineering, College of Engineering, Qatar University, Doha 2713, Qatar; m.Khraisheh@qu.edu.qa; 8 Department of Zoology, The Islamia University of Bahawalpur, Bahawalpur 63100, Pakistan; mushtaqlashary@gmail.com

**Keywords:** separation factor, inorganic filler, composite AEM, acid recovery, ion exchange capacity, diffusion dialysis

## Abstract

In this work, the synthesis of a series of the functionalized inorganic/organic composite anion exchange membranes (AEMs) was carried out by employing the varying amount of inorganic filler consist of *N*-(trimethoxysilylpropyl)-*N*,*N*,*N*-trimethylammonium chloride (TMSP-TMA^+^Cl^−^) into the quaternized poly (2, 6-dimethyl-1, 4-phenylene oxide) (QPPO) matrix for acid recovery via diffusion dialysis (DD) process. Fourier transform infrared (FTIR) spectroscopy clearly demonstrated the fabrication of the functionalized inorganic/organic composite AEMs and the subsequent membrane characteristic measurements such as ion exchange capacity (IEC), linear swelling ratio (LSR), and water uptake (W_R_) gave us the optimum loading condition of the filler without undesirable filler particle aggregation. These composite AEMs exhibited IEC of 2.18 to 2.29 meq/g, LSR of 13.33 to 18.52%, and W_R_ of 46.11 to 81.66% with sufficient thermal, chemical, and mechanical stability. The diffusion dialysis (DD) test for acid recovery from artificial acid wastewater of HCl/FeCl_2_ showed high acid DD coefficient (U_H_^+^) (0.022 to 0.025 m/h) and high separation factor (S) (139-260) compared with the commercial membrane. Furthermore, the developed AEMs was acceptably stable (weight loss < 20%) in the acid wastewater at 60 °C as an accelerated severe condition for 2 weeks. These results clearly indicated that the developed AEMs have sufficient potential for acid recovery application by DD process.

## 1. Introduction

Water pollution is going to be a severe risk to our environment and the availability of clean water assets with the continuous increase in urbanization and industrialization. Industries such as steel processing, mining, ion exchange resin regeneration, metallurgical and electroplating discharge acidic wastewater without any pretreatment [1,2,3]. This polluted water possesses a huge quantity of metal salts and acids that largely exceed the safety level which surrenders to polluting the environment and also deteriorate the equipment [1,2,4,5,6]. Solvent extraction, neutralization, crystallization, thermal decomposition, and coagulation-flocculation are the commonly employed methods for treating acidic wastewater. The heating and addition of alkaline solution are required by all these methods and approaches, which demands high energy expenditure and significantly further pollutes the environment by the discharge of chemicals/salts [7,8,9].

Membrane technology compared to other separation technologies by employing anion exchange membranes (AEMs) is favorable for waste acid recovery via diffusion dialysis (DD) process. It is an emanating technology for acid recovery, which is established on the difference in diffusivity between salts and acids [10]. Especially, the AEM-based DD process is attracting remarkable attention currently to recover acid from the acid drainage because of its easy installation protocol, lower energy consumption, and less operating cost [11]. Moreover, it is a thermodynamically good technology as it involves a decline in over-all Gibbs free energy and increase in entropy of the system [12]. It has been largely used in the recovery of sulphuric acid [13], hydrochloric acid [14], nitric acid [15], and a mixed acid [16,17]. In this process, metal ions, proton, and counter ions transfer informally from their higher concentration compartment to the lower concentration compartment because of the mass transfer concentration gradient across the ion exchange membranes (IEM) [12,18,19]. The AEMs with fixed cationic groups ease the transport of anions through the membranes by adsorbing their counter-ions with lower charge number and smaller hydration radius and some divalent or high-valence metal ions would be eliminated because of their relatively larger hydrated radius in order to balance the charges [7,14,20]. Therefore, generally, the clear transparent acid was obtained in the lower concentration side and the purity of recovered acid was further increased [7,20]. The separation of acid from salts is possible through a positively charged AEMs, which permits the selective migration of anions across the membrane while remaining impenetrable to cations rather than protons mature to their small size and weak positive charge [12]. 

The AEM has received extraordinary interest as an important part of DD process [18,21]. It must exhibit reasonable water uptake, good thermo-mechanical stability, excellent chemical resistance, and high anion permeability and selectivity along with a low price for DD process [22]. Usually, they were prepared by bonding ion-exchange groups with a polymeric material [23,24]. On the properties of the AEM, the polymeric material has an important effect as stability and selectivity are dependent on the hydrophobicity and mechanical strength. To achieve mandatory IEC for the AEM, advanced well-connected ion-conducting channels are also required which will increase the DD performance as IEC represents a crucial role in the selectivity of ions [25,26]. The lower selectivity between proton (H^+^) and other cations is resulted by bonding of ion-exchange group to the polymeric material. Moreover, the AEMs with higher IEC represented higher swelling degree and modest mechanical stability. To date, several polymeric materials namely polysulfone [27], poly(tetrafluoroethylene) [28], polyvinyl alcohol (PVA) [29], chitosan [30], polybenzimidazole [31], and bromo-methylated poly (phenylene oxide) (BPPO) [32,33,34], etc. were utilized for the development of the AEMs. Herein, poly (2,6-dimethyl-1,4-phenylene oxide) (PPO) was largely utilized as polymeric material for the preparation of AEMs due to excellent membrane forming ability and mechanical properties. Bromination of PPO provides brominated poly (2,6-dimethyl-1,4-phenylene oxide) (BPPO) that acts as a precursor for AEMs. Due to the presence of a highly reactive –CH_2_Br group, BPPO contains outstanding membrane formation and functionalizable properties [26,35]. 

Inorganic filler such as *N*-(trimethoxysilylpropyl)-*N*,*N*,*N*-trimethylammonium chloride is crucial for the development of the functionalized inorganic/organic composite AEMs by incorporating into the quaternized poly (2,6-dimethyl-1,4-phenylene oxide) (QPPO) matrix. It is functionalized inorganic filler that will enhance the conductivity of the prepared functionalized inorganic/organic composite AEMs. It was our first attempt to employ it as an inorganic filler to prepare the functionalized inorganic/organic composite AEMs for acid recovery application according to the best of our knowledge. The low acidity of trimethylamine (PK_a_ = 9.81) permits the higher permeability of anions because they are dissociated from the functional groups [18,36]. The prepared functionalized inorganic/organic composite AEMs will contain a trimethylamine group responsible for higher permeability of anions, leading to higher acid recovery efficiency of the prepared composite AEMs.

Previously, we reported the synthesis of polymeric AEMs by introducing different ion-exchange groups into the polymeric material for adsorption [37], diffusion dialysis [26,36], electrodialysis [33,34,38], and alkaline fuel cell [39] applications. Herein, we reported the fabrication of a series of the functionalized inorganic/organic composite AEMs by incorporating the varying amount of *N*-(trimethoxysilylpropyl)-*N*,*N*,*N*-trimethylammonium chloride into the QPPO matrix via the solution casting method. FTIR test confirmed the successful synthesis of the composite AEMs. They were characterized in terms of water uptake, ion exchange capacity, linear swelling ratio, morphology, acidic, mechanical, and thermal stability. They were used for acid recovery by using the simulated mixture of HCl/FeCl_2_ as a model feed solution via DD process at ambient temperature. The acid recovery efficiency of the composite AEMs was compared with commercial membrane DF-120B and already reported AEMs in literature at ambient temperature. 

## 2. Experimental

### 2.1. Materials

Sodium carbonate (Na_2_CO_3_), hydrochloric acid (HCl), trimethylamine, *N*-(trimethoxysilylpropyl)-*N*,*N*,*N*-trimethylammonium chloride (TMSP-TMA^+^Cl^−^), N-Methyl-2-pyrrolidone (NMP), dimethyl sulfoxide (DMSO), ferrous chloride (FeCl_2_. 4H_2_O), sodium sulfate (Na_2_SO_4_), and potassium permanganate (KMnO_4_) were purchased from Sinopharm Chemical Reagent Co. Ltd., Shanghai, China and used as received. Tianwei Membrane Co. Ltd. Shandong of China kindly supplied the brominated poly (2, 6-dimethyl-1, 4-phenyleneoxide) (BPPO). Throughout this research, deionized water was utilized. 

### 2.2. Preparation of the Quaternized poly (2,6-dimethyl-1,4-phenylene Oxide) Membrane

**Figure 1:**
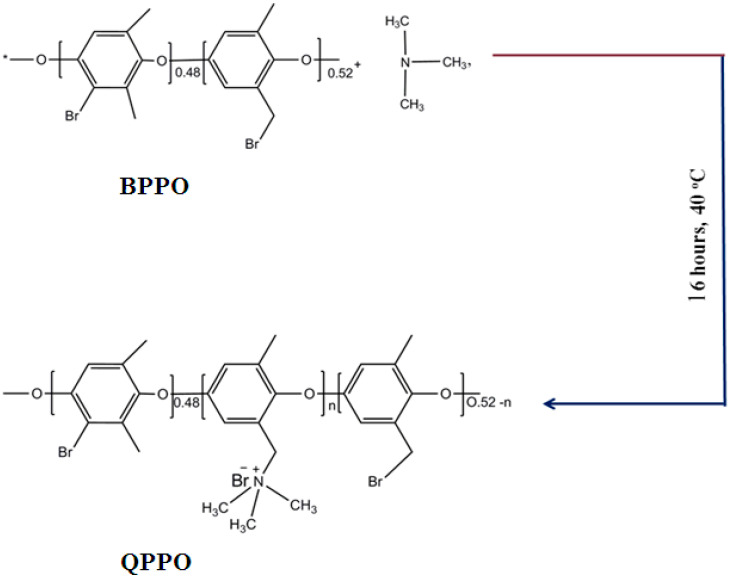
Fabrication of the quaternized poly (2,6-dimethyl-1,4-phenylene oxide) (QPPO) anion exchange membrane (AEM).

Quaternization of PPO was performed as described (Figure 1) [38,40]. In a typical method, 0.8 g of BPPO was dissolved into N-Methyl-2-pyrrolidone (NMP) solvent to get a homogeneous solution. After that, the measured amount of trimethylamine (0.125 g) was added to the prepared BPPO solution. The resultant mixture was stirred at 40 °C for 16 h and then casted onto the hot glass plate at 60 °C for 24 h. The attained membrane was washed and stored in deionized (DI) water.

Moreover, the QPPO powder was also attained by precipitating the mixture of BPPO and trimethylamine after stirring at 40 °C for 16 h into ethanol for the preparation of functionalized inorganic/organic composite AEMs. It was dried in a vacuum oven at 40 °C for 2 days. The attained QPPO powder was utilized for the preparation of composite AEMs.

### 2.3. Preparation of the Functionalized Inorganic/Organic Composite AEMs

We utilized solution casting method to synthesize the functionalized inorganic/organic composite AEMs as reported in our previous research [26,36,37,39]. Initially, the quaternized poly (2,6-dimethyl-1,4-phenylene oxide) (QPPO) was dissolved into dimethyl sulfoxide (DMSO). Then 0.025, 0.05, 0.075, and 0.10 g of *N*-(trimethoxysilylpropyl)-*N*,*N*,*N*-trimethylammonium chloride was added into solution of QPPO. The mixture was stirred for 5 h and then casted onto the glass plate and heated (solvent evaporation) for one day at 60 °C. The prepared functionalized inorganic/organic composite AEMs were designed as QPPO-2.5, QPPO-5, QPPO-7.5, and QPPO-10, respectively, where 2.5, 5, 7.5, and 10 refer to the weight percentage (%) of N-(trimethoxysilylpropyl)-*N*,*N*,*N*-trimethylammonium chloride into the QPPO. They were peeled off from the glass plates and cleaned with distilled water prior to study and characterization.

### 2.4. Characterizations

#### 2.4.1. Instrumentations

FTIR analysis of the prepared functionalized inorganic/organic composite AEMs was carried out by employing FTIR spectrometer (Vector 22, Bruker, MA, USA) having a resolution of 2 cm^−1^ and a total spectral range of 4000–400 cm^−1^. Mechanical stability of hydrates membranes was measured by employing Q800 dynamic mechanical analyzer (DMA, TA Instruments, Kyoto, Japan) at a stretch rate of 0.5 N/min. Mechanical stability of the composite AEMs was investigated in wet state. Thermal stability of the prepared composite AEMs was investigated by employing a Shimadzu TGA-50H analyzer (Shimadzu Corporation, Kyoto, Japan) within the temperature range of 25 °C to 800 °C under nitrogen flow, with a heating rate of 10 °C/min. The detailed morphology of the prepared composite AEMs was investigated through a field emission scanning electron microscope (FE-SEM, Sirion200, FEI Company, Hillsboro, OR, USA). The samples of surfaces and cross-sections of the composite AEMs were taken in a dry state.

#### 2.4.2. Water Uptake and Linear Swelling Ratio

Water uptake (*W*_R_) was measured to study hydrophilicity of the IEMs. It has a crucial effect on the DD performance of AEMs. A suitable range of water uptake is essential for DD application. The functionalized inorganic/organic composite AEMs were oven-dried and then accurately weighed to determine dry weight. They were soaked into deionized water at 25 °C for 24 h. The wet weight was noted after the removal of surface water with absorbing paper. It was estimated from the difference in weight before and after complete drying the composite AEMs by using the following relationship:

(1) $$W_{R}=\frac{W_{WET}-W_{DRY}}{W_{DRY}}\times 100\%$$

where *W_DRY_* and *W_WET_* are the weights of dry and wet functionalized inorganic/organic composite AEMs, respectively.

The dimensional stability of the prepared composite AEMs has a significant influence on the DD application. To achieve higher DD efficiency, proper dimensional stability is required. For the functionalized inorganic/organic composite AEMs, linear swelling ratio (*LSR*) was investigated by soaking into deionized water at 25 °C. For it, they were cut into (2 × 2) cm^2^ pieces. It was measured by using below equation [41]:

(2) $$LSR=\frac{L_{WET}-L_{DRY}}{L_{DRY}}\times 100\%$$

where *L_DRY_* and *L_WET_* are the lengths of dry and wet functionalized inorganic/organic composite AEMs, respectively.

#### 2.4.3. Ion Exchange Capacity

Ion exchange capacity (IEC) denotes the number of exchangeable ionic groups (equivalents) available per dry weight of IEM [34]. The classical Mohr method was used to calculate IEC of the prepared functionalized inorganic/organic composite AEMs as reported in our previous work [26,36,39]. For calculation of IEC, the prepared functionalized inorganic/organic composite AEMs were initially converted into the chloride ion (Cl^−^) form by soaking into 1.0 M NaCl solution for 2 days. To discharge excess quantity of NaCl, they were cleaned with distilled water very carefully. They were then soaked for 2 days into 0.5 M Na_2_SO_4_ solution. The titration method was employed to estimate liberated quantity of Cl^−^ ions. It was carried out by using 0.05 (M) AgNO_3_ as a titrant and K_2_CrO_4_ as an indicator. It was measured by using below relationship:

(3) $$IEC=\frac{VC}{m}$$

where *m*, *V*, and *C* represent the dry mass of the functionalized inorganic/organic composite AEMs, titer volume during titration, and the concentration of AgNO_3_ solution, respectively.

Depending on the water uptake and *IEC*, the numbers of bound water molecules per ionic group (*λ*) were calculated by the following equation:

(4) $$\lambda =\frac{W_{WET}-W_{DRY}\times 1000}{IEC\times W_{DRY}\times 18}$$

#### 2.4.4. Acidic Stability Test

The acidic stability of AEMs was used to determine their life time in DD application. The acidic stability of the prepared functionalized inorganic/organic composite AEMs was revealed in terms of weight loss by soaking into the mixture of feed solution (the mixture of HCl/FeCl_2_) at 60 °C for two weeks. 

#### 2.4.5. Diffusion Dialysis Test

**Figure 2:**
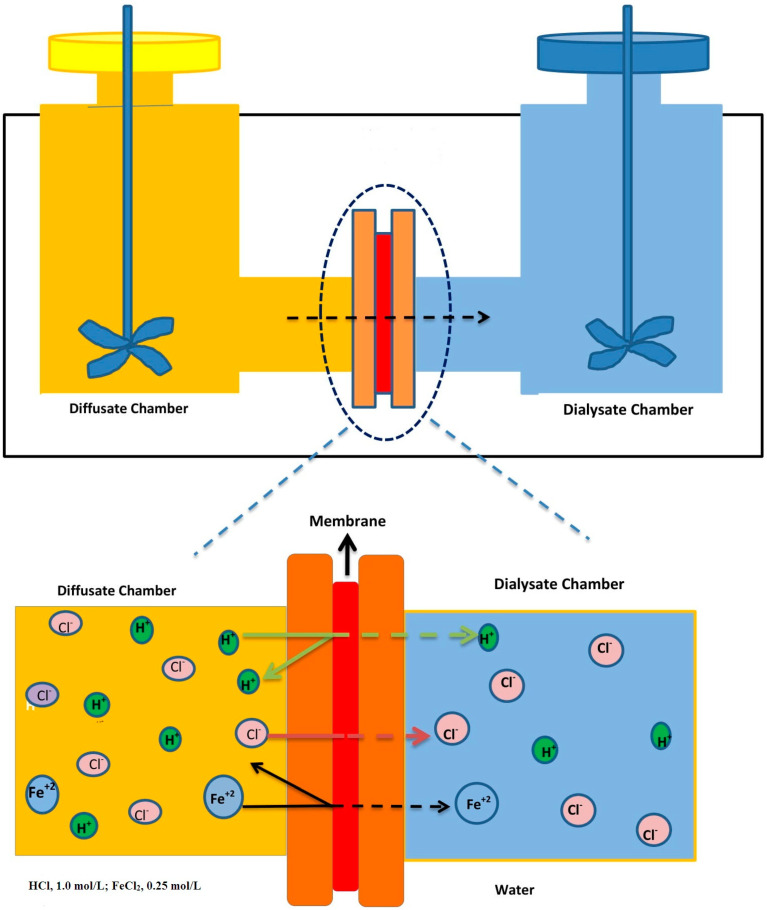
Schematic principle of the diffusion dialysis (DD) process for acid recovery from the simulated mixture of HCl/FeCl_2_.

Herein, the DD test was used to measure the acid recovery capability of the prepared composite AEMs as reported in literature [26,36,42]. In this research, we used a two-compartment cell (Figure 2). Both compartments were separated by utilizing the functionalized inorganic/organic composite AEM with an effective area of 5.7 cm^2^, as shown in Figure 2. Before the DD test, the prepared composite AEMs were conditioned very carefully for 5 h into the feed solution (0.81 M HCl + 0.18 M FeCl_2_). Then, they were washed with distilled water. During this test, one compartment of the cell was filled up with 100 mL feed solution, whereas the other side with 100 mL distilled water. Both sides were stirred vigorously in order to minimize the concentration polarization. This test for each composite AEM was carried out for one hour. Lastly, both solutions (feed and permeate) were taken from different compartments. The concentration of H^+^ in both compartments was determined by titration with Na_2_CO_3_ aqueous solution, whereas FeCl_2_ concentration was determined by titration with KMnO_4_ aqueous solution. 

The below relationship was used to measure the DD coefficients (*U*) for the composite AEMs [26]:

(5) $$U=\frac{M}{At\Delta C}$$

where *A* is the effective membrane area (m^2^), M is the amount of component transported in (mol), ∆*C* is the logarithm average concentration between the two chambers (mol/m^3^), *t* is the time (h), and ∆*C* was measured by employing below relationship [26]:

(6) $$\Delta C=\frac{C_{f}^{0}-(C_{f}^{t}-C_{d}^{t})}{\ln[C_{f}^{0}/(C_{f}^{t}-C_{d}^{t})]}$$

where $C_{f}^{0}$ and $C_{f}^{t}$ are feed concentrations at time 0 and *t*, respectively, and $C_{d}^{t}$ is the dialysate.

The DD coefficient of acid (*U_H_*) and metal (*U_Fe_*) can be measured based on Equations (5) and (6). The separation factor (*S*) is the ratio of DD coefficients (*U*) of the two species in the solution and can be measured by using the below relationship [36]:

(7) $$S=\frac{U_{H}}{U_{Fe}}$$

## 3. Results and Discussions

### 3.1. FTIR

**Figure 3:**
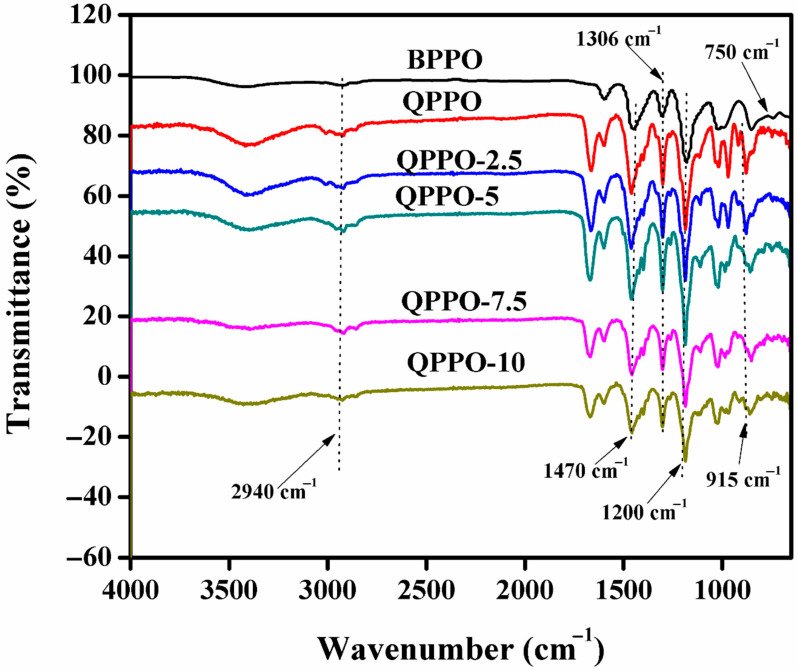
FTIR spectra of the pure BPPO membrane, QPPO membrane, and functionalized inorganic/organic composite AEMs QPPO-2.5 to QPPO-10.

In this research, FTIR test was used to demonstrate the quaternization of PPO and the fabrication of the functionalized inorganic/organic composite AEMs. FTIR spectra of pure BPPO membrane, QPPO membrane, and the prepared inorganic/organic composite AEMs QPPO-2.5 to QPPO-10 are represented in Figure 3. The peak at 750 cm^−1^ was due to C-Br stretching vibration in the pristine BPPO membrane [36,43]. The symmetrical and asymmetrical stretching of C–O are at 1200 and 1306 cm^–1^, and those of phenyl groups at 1470 and 1600 cm^–1^, respectively [36,44]. The bands at 1446 cm^–1^ were attributed to –CH stretching (V and δ) [36]. The peak at 750 cm^−1^ due to C-Br was disappeared in the spectra of QPPO membrane and the composite AEMs, indicating the successful quaternization of PPO [42,45]. In the QPPO and the prepared composite AEMs, the peak at 915 cm^−1^ was associated with C-N stretching vibration. It was absent in spectra of the pure BPPO membrane. The peak at 2940 cm^−1^ becomes broader due to the linkage of methyl group (-CH_3_) containing trimethylamine to the BPPO polymeric material via quaternization reaction and introduction of *N*-(trimethoxysilylpropyl)-*N*,*N*,*N*-trimethylammonium chloride into the QPPO. It demonstrated the successful fabrication of QPPO membrane and the composite AEMs.

### 3.2. Morphology Test

**Figure 4:**
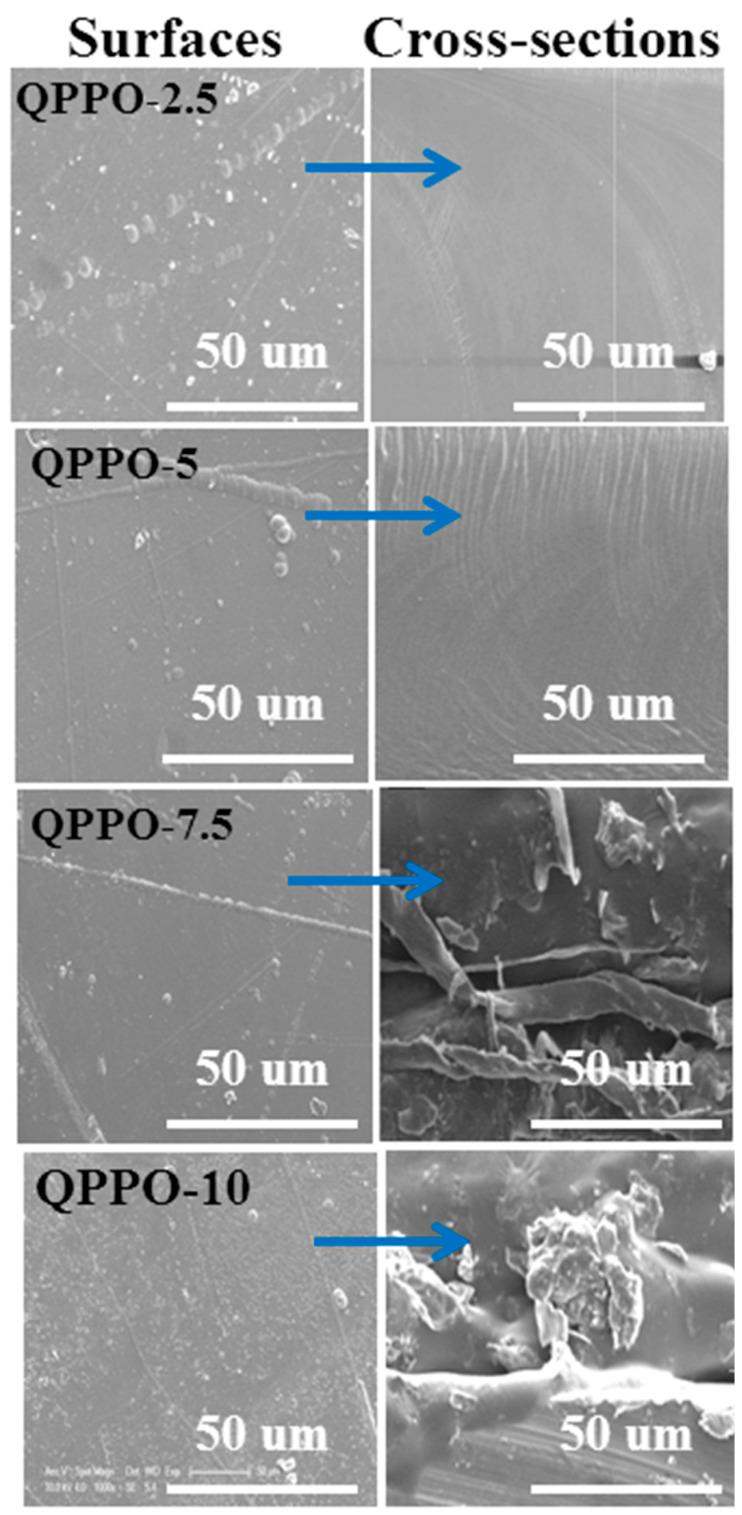
SEM micrographs of surfaces and cross-sections of the prepared functionalized inorganic/organic composite AEMs.

Morphology of IEMs has a crucial influence on the transport properties. For the prepared functionalized inorganic/organic composite AEMs, the structural features were investigated by using a Field emission scanning electron microscope (FE-SEM, Sirion200, FEI Company, Hillsboro, OR, USA). Figure 4 represents SEM micrographs of surfaces and cross-sections of the prepared composite AEMs. Results represent that there were no obvious particles into the surfaces as well as cross-sections of the prepared composite AEMs when the concentration of *N-*(trimethoxysilylpropyl)-*N*,*N*,*N*-trimethylammonium chloride was in the range of 2.5 to 5.0% into the QPPO, indicating the good dispersion of it. Nevertheless, its excessive incorporation into the QPPO matrix showed particle aggregation. From Figure 4, it was noted that when the amount of inorganic filler into the QPPO was enhanced higher than 5% (7.5 or 10%). Then, the particle agglomeration occurred which results in the decline in the selectivity of the composite AEMs because of the voids formed between the polymeric material and the inorganic phase. With increasing the amount of *N*-(trimethoxysilylpropyl)-*N*,*N*,*N*-trimethylammonium chloride to 10% into the QPPO, the aggregation of particles occurred which results in a decrease in acid recovery efficiency of the composite AEM QPPO-10. Therefore, its suitable amount should be selected to achieve higher DD performance.

### 3.3. Ion Exchange Capacity

**Figure 5:**
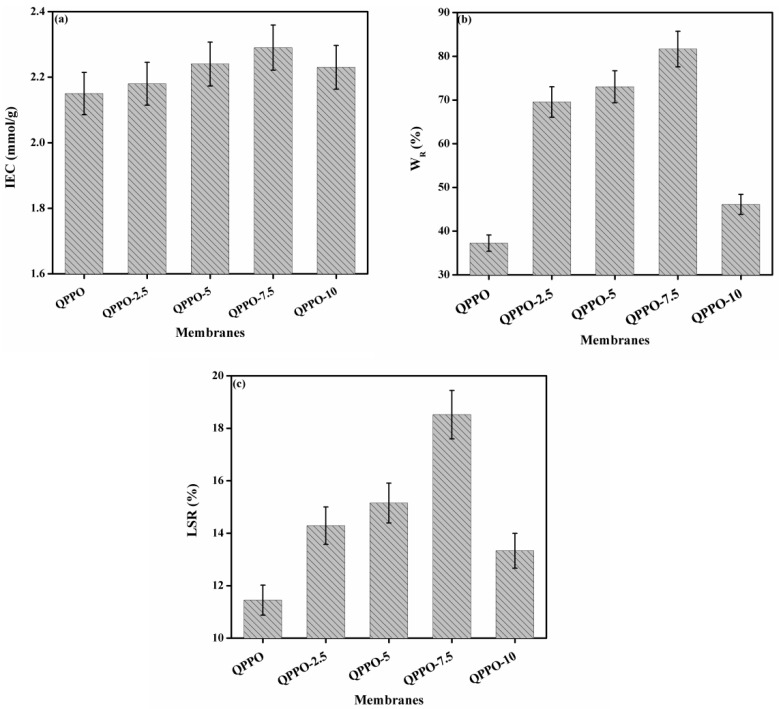
(**a**) Ion exchange capacity, (**b**) water uptake, (**c**) linear swelling ratio of the prepared functionalized inorganic/organic composite AEMs at room temperature.

Ion exchange capacity is a crucial property of IEMs utilized in electrochemical processes. It provides useful information about the charge density of IEMs and it is also a significant parameter that governs conductivity and transport properties of IEMs [40]. It depends on the amount of ion exchange groups connected to the polymeric material and is one of the significant properties of AEM due to the direct relevance of anion permeability [18]. For the prepared QPPO membrane and the composite AEMs, the classical Mohr’s method was employed to determine it, and attained results are denoted in Figure 5a. Its value for QPPO membrane was found to be 2.15 meq/g. For the prepared composite AEMs QPPO-2.5 to QPPO-10, it was found to be 2.18 to 2.29 meq/g. It was found to be increased with increasing the amount of inorganic filler into the QPPO from 2.5% to 7.5% whereas decreased with further increasing its amount into the QPPO (up to 7.5%). Thus, the prepared composite AEMs were highly charged which are useful for DD applications.

### 3.4. Water Uptake and Linear Swelling Ratio

Water uptake has a significant influence on the electrochemical application of IEMs, especially on DD process. For the AEM, it is of great significance as a bridge to balance ion permeability and mechanical properties [18]. It is well established that it plays an important role in the migration of ions, and moderate water uptake is required for high anion permeability [18]. Figure 5b shows water uptake of QPPO membrane as well as the prepared composite AEMs as a function of the amount of inorganic filler into the QPPO. It was found to be 37.25% for the QPPO membrane at ambient temperature. For the prepared composite AEMs QPPO-2.5 to QPPO-10, it was found to be increased from 69.54% to 81.67% with increasing the amount of inorganic filler from 2.5% to 7.5% into the QPPO whereas decreased with further increasing its amount (up to 7.5%) into QPPO due to the aggregation of structure of the polymeric AEMs. It was due to existence of same functional group (–N(CH3)^+^Cl^−^) into QPPO as well as of *N*-(trimethoxysilylpropyl)-*N*,*N*,*N*-trimethylammonium chloride.

To investigate the influence of IEC on water uptake of the prepared composite AEMs, the number of water molecules absorbed by each ion-conducting group (λ) was measured and is given in Table 1. From here, it was noted that the number of water molecules absorbed by each ion-conducting group increased with increasing the amount of inorganic filler from 2.5% to 7.5% into QPPPO matrix from QPPO-2.5 to QPPO-7.5 because water uptake and IEC were also increased. It was decreased with a further increasing amount of inorganic filler (up to 7.5) into QPPO matrix due to a decrease in water uptake and IEC.

For DD application, linear swelling ratio (dimensional stability) of IEMs is a crucial parameter. A higher swelling ratio results in the excessive permeation of ferrous ion (Fe^2+^) through the AEMs which leads to a lower separation factor. Figure 5c indicates a linear swelling ratio of QPPO membrane and the prepared composited AEMs at ambient temperature. The value of the linear swelling ratio for QPPO membrane was 11.50%. For the prepared composite AEMs QPPO-2.5 to QPPO-10, its value was increased from 14.29–18.52% by increasing the quantity of inorganic filler into the QPPO. It was ideal for AEM employed for acid recovery via DD process. Higher dimensional stability of the prepared composite AEMs was based on BPPO polymeric material. Therefore, the prepared functionalized inorganic/organic composite AEMs exhibited good swelling resistance. Hence, it is mandatory for their long time running during acid recovery application. 

### 3.5. Mechanical and Thermal Stability

For a long time running in acid recovery application, the higher mechanical stability of AEMs is also crucial. Mechanical stability of QPPO membrane and the prepared composite AEMs was determined in wet state by using a dynamic mechanical analyzer (DMA). Tensile strength (TS) represents resistance to mechanical force of the membranes, whereas elongation at break (E_b_) indicates the flexibility of the IEMs. Table 1 shows the values of tensile strength (TS) and elongation at break (E_b_) of the QPPO membrane as well as composite AEMs. The value of TS for QPPO membrane was 19 MPa and E_b_ was 10%. For the prepared composite AEMs, the value of TS was in the range of 22 to 48 MPa, whereas E_b_ was in the range of 14% to 56%. Results show that the prepared composite AEM QPPO-7.5 showed higher flexibility among the prepared composite AEMs due to the higher value of E_b_. From Table 1, it was noted that the value TS followed the same order as IEC and water uptake of the prepared composite AEMs with increasing the amount of inorganic filler into the QPPO. The value of E_b_ of the prepared composite AEMs in the range of 14% to 56% was lower than previously reported AEMs [46]. On the contrary, the value TS of the prepared composite AEMs in the range of 22 to 48 MPa was higher than previously reported work [47], indicating that the prepared composite AEMs exhibited higher mechanical stability necessary for DD application. Similar results are reported in the literature that BPPO-based IEMs showed higher mechanical stability [48,49].

**Figure 6:**
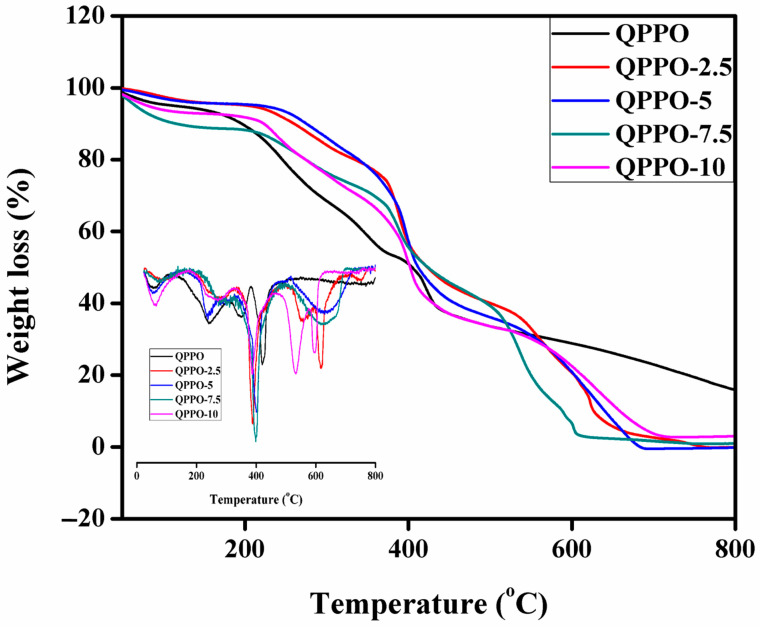
TGA thermograms as well as onset differential thermogravimetric analysis (DrTGA) of the QPPO and prepared functionalized inorganic/organic composite AEMs QPPO-2.5 to QPPO-10.

Generally, BPPO-based IEMs exhibited excellent thermal stability as reported in literature [48,50]. It is associated with the hydrophobic nature of BPPO polymeric material. Thermal stability is a significant parameter of IEMs. Thermal stability of the QPPO membrane and prepared composite AEMs was investigated by thermogravimetric analyzer (TGA) under a constant nitrogen flow at a constant heating rate of 10 °C/min within the temperature range from 30 to 800 °C. Figure 6 represents TGA thermographs of the QPPO and composite AEMs. It was noted that the weight loss took place in three consecutive stages for the QPPO membrane and prepared composite AEMs. The first weight loss stage was observed below 130 °C. It was associated with the evaporation of residual water and solvent [26,40]. The second weight loss was noted in the range of 180 to 240 °C. It was due to the degradation quaternary ammonium group into the polymeric material [32,33]. The final weight loss was found around 470 °C. It was due to the degradation of polymeric material [39,51]. Moreover, we also received the same results from differential thermogravimetric analysis (DrTGA) graphs (Figure 6 onset) as confirmed in the above discussion. The first weight loss was associated with water loss from the membrane matrix, whereas the second weight loss was due to degradation of quaternary ammonium groups into the polymer matrix. The final weight loss was attributed to degradation of the polymeric material. It was concluded that the prepared composite AEM QPPO-2.5 showed higher thermal stability, whereas QPPPO-7.5 exhibited the lowest thermal stability among the prepared composite membranes. Thermal stability of the composite AEMs decreased from membrane QPPO-2.5 to QPPO-7.5. Overall, the prepared composite AEMs showed good thermal stability mandatory for DD process.

### 3.6. Acid Recovery Performance

**Figure 7:**
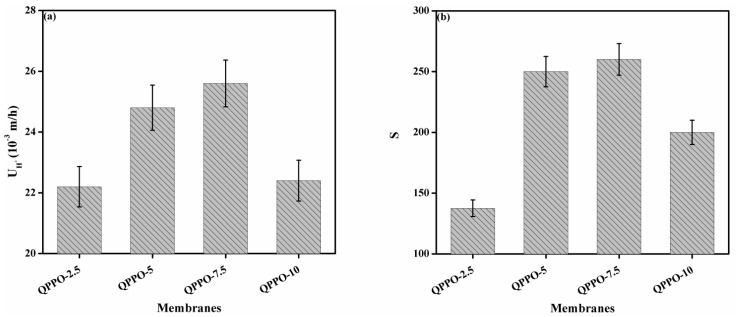
(**a**) The DD coefficient of acid (U_H_^+^), (**b**) separation factor (S) of the prepared functionalized inorganic/organic composite AEMs at room temperature.

**Figure 8:**
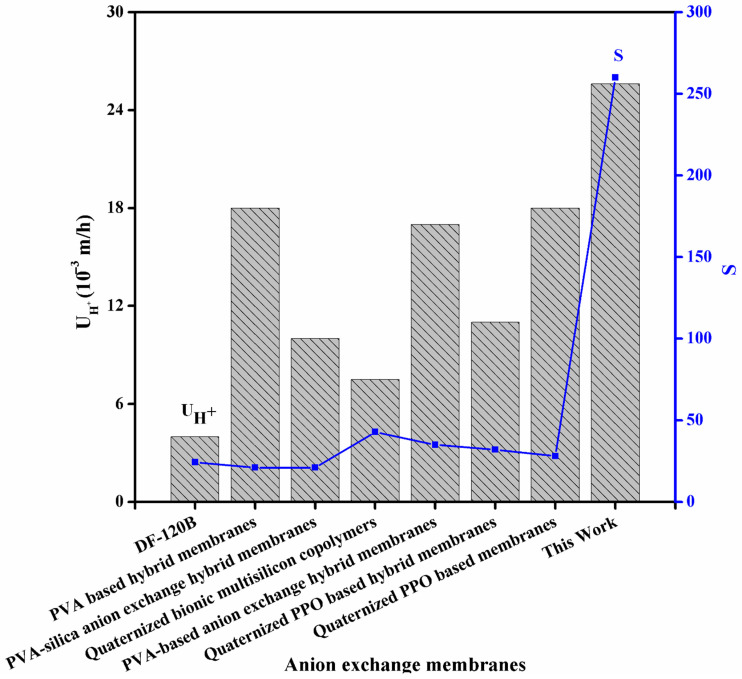
Comparison of the DD efficiency (diffusion dialysis coefficient of acid (U_H_^+^) and separation factor (S)) of the prepared functionalized inorganic/organic composite AEM (This work) with the reported AEMs in literature at room temperature [9,32,42,44,52,53,54].

Figure 7a shows the acid recovery performance of the prepared functionalized inorganic/organic composite AEMs at room temperature. The acid recovery performance was evaluated in batch mode via DD process. This test was performed by utilizing the simulated mixture of HCl/FeCl_2_ as a model feed. At room temperature, the value of DD coefficient of acid (U_H_^+^) was found to be 20 to 25.6 (10^−3^ m/h) for the prepared composite AEMs. Results indicate that the value of DD coefficient of acid (U_H_^+^) was higher than commercial membranes DF-120B (U_H_^+^ = 4×10^−3^ m/h) [26,42]. From the attained results, it was observed that the value of DD coefficient of acid was found to be increased from 20 to 25.6 (10^−3^ m/h) with increasing the amount of inorganic filler from 0.025 to 0.75 g into the QPPO. Figure 8 provides an interesting comparison of the fabricated composite AEMs with the membranes reported in the literature. From here, we conclude that the prepared composite AEMs are outstanding candidates for acid recovery via DD process. It was due to an increase in water uptake and IEC of the fabricated composite AEMs with increasing the amount of inorganic filler into the QPPO. It results in the increase in migration of hydrogen ion (H^+^) through the prepared composite AEMs, which leads to an increase in the value of DD coefficient of acid. Contrary, with increasing the amount of inorganic filler into the QPPO (up to 7.5%), the value of DD coefficient of acid was found to be decreased from 25.6 to 22.4 (10^−3^ m/h). It was due to a decline in hydrophilicity of the prepared composite AEM. From here, we concluded that the prepared composite AEMs exhibited higher acid recovery when the amount of inorganic filler into the QPPO was 7.5%. After this amount, the value of DD coefficient of acid (U_H_^+^) was decreased. It denotes that the prepared composite AEMs exhibited outstanding potential for acid recovery via DD process.

The separation factor (S) is defined as the ratio of U_H_^+^ to U_Fe_^2+^. Figure 7b depicts the separation factor of the fabricated functionalized inorganic/organic composite AEMs at ambient temperature. The obtained value of separation factor was increased from 139 to 260 with increasing the amount of inorganic filler from 2.5% to 7.5% into the QPPO, which surpassed the evaluated commercial membrane DF-120B (24.3) [32,42]. With increasing the amount of inorganic filler, water uptake, linear swelling ratio (dimensional stability), and IEC were increased. The increase in swelling ratio (Figure 5c) was not higher due to the usage of BPPO as polymeric material which made the prepared composite AEMs suitable for the DD application. A higher increase in swelling leads to lower selectivity of membranes. The obtained higher value of separation factor (139 to 260) was due to the lower swelling of the prepared composite AEMs. It showed that the attained value of swelling (14.29 to 18.52%) has no negative effect on the selectivity of the composite AEMs. With increasing IEC, more ion-exchange groups were available into the prepared composite AEMs. Therefore, the increase in IEC was crucial for higher acid recovery performance. On the contrary, the hydrophilicity of the prepared composite AEMs was decreased with further increasing the amount of inorganic filler (up to 7.5%) into the QPPO, which results in the decrease in the value of the separation factor. Hence, the appropriate quantity of inorganic filler was required to attain higher acid recovery performance of composite AEMs via DD process.

### 3.7. Acid Stability

**Figure 9:**
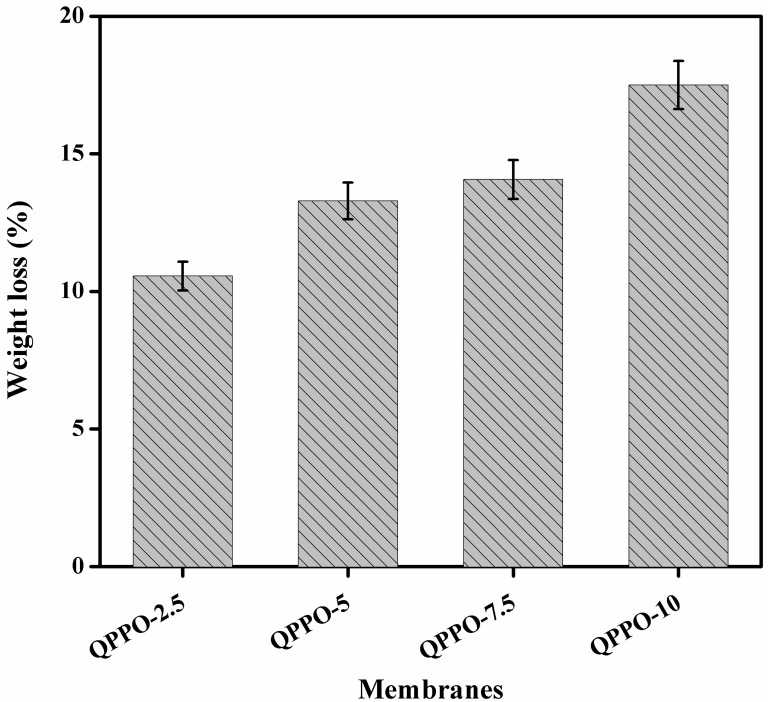
Weight loss of the prepared functionalized inorganic/organic composite AEMs measured after soaking into the mixture of HCl/FeCl_2_ feed solution for 2 weeks at 60 °C, indicating excellent acid stability.

The acid stability was investigated in terms of weight loss of the prepared inorganic/organic composite AEMs by soaking into the mixture of HCl/FeCl_2_ feed solution at 60 °C for 2 weeks. Figure 9 represents the weight loss of the prepared composite AEMs measured after 2 weeks. Results represent that the weight loss was increased from 11% to 18% for the composite AEMs QPPO-2.5 to QPPO-10. The weight loss was increased with increasing the amount of inorganic filler from membrane QPPO-2.5 to QPPO-10. The maximum weight loss was only 18% after 2 weeks. The color of membranes was unchanged. From this, we conclude that the prepared composite AEMs represented excellent acid stability. Therefore, they are useful for acid recovery via DD process for a long time.

## 4. Conclusions

In this article, a series of functionalized inorganic/organic composite AEMs were fabricated via the solution casting method. The successful fabrication of composite AEMs was demonstrated by employing FTIR spectroscopy. They showed extraordinary mechanical, thermal, acid stability. The effect of the amount of inorganic filler on the physico-chemical properties was revealed in detail. They represented IEC of 2.18 to 2.29 meq/g, water uptake of 46.11% to 81.66%, and linear swelling ratio of 13.33% to 18.52%. For the prepared composite AEMs, the DD coefficient of acid (U_H_^+^) was in the range of 0.022 to 0.025 m/h, whereas the separation factor (S) was in the range of 139 to 260 at ambient temperature. It demonstrated that the prepared functionalized inorganic/organic composite AEMs are outstanding candidates for acid recovery application at room temperature.

## Figures and Tables

**Table 1:** Tensile strength, elongation at break, hydration number and thickness of QPPO, and the prepared functionalized inorganic/organic composite AEMs QPPO-2.5 to QPPO-10.

Membranes	TS (MPa)	E_b_ (%)	λ	Thickness (μm)
QPPO	19 ± 0.95	10 ± 0.50	10.40 ± 0.52	62 ± 3.10
QPPO-2.5	48 ± 2.40	27 ± 1.35	17.72 ± 0.87	67 ± 3.35
QPPO-5	39 ± 1.95	41 ± 2.10	18.11 ± 0.91	61 ± 3.10
QPPO-7.5	32 ± 1.60	56 ± 2.80	19.80 ± 1.00	73 ± 3.65
QPPO-10	22 ± 1.10	14 ± 0.70	11.50 ± 0.58	59 ± 2.95

## Data Availability

The data presented in this study is available on request from the corresponding author.

## Nomenclature

Codes	Full names
AEM	Anion exchange membrane
BPPO	Brominated poly(2,6-dimethyl-1,4-phenylene oxide)
DD	Diffusion dialysis
TMSP-TMA^+^Cl^−^	*N*-(trimethoxysilylpropyl)-*N*,*N*,*N*-trimethylammonium chloride
IEC	Ion exchange capacity
IEM	Ion exchange membrane
